# Effects of strigolactone on photosynthetic and physiological characteristics in salt-stressed rice seedlings

**DOI:** 10.1038/s41598-020-63352-6

**Published:** 2020-04-10

**Authors:** Fenglou Ling, Qingwang Su, Hao Jiang, Jingjing Cui, Xiaoliang He, Zhihai Wu, Zhian Zhang, Juan Liu, Yongjun Zhao

**Affiliations:** 1 Agronomy College, Jilin Agricultural University, Changchun, 130118 Jilin China; 2 Jilin Danong Seed Industry Co., Ltd., Changchun, 130118 Jilin China; 3 College of Biological, Chemical Science and Engineering, Jiaxing University, Jiaxing, 314001 Zhejiang China

**Keywords:** Plant stress responses, Plant breeding, Strigolactone

## Abstract

Saline stress has been identified as the primary factor inhibiting rice seedling growth, which represents a complex abiotic stress process. Most plant hormones have been shown to alleviate the plant damage caused by salt stress. The effects of synthetic strigolactone (GR24) on Jinongda 667 rice seedlings treated with 200 mM NaCl were studied. Photosynthesis and its related physiological characteristics were analyzed in salt-stressed rice seedlings treated with GR24. NaCL stress inhibited the growth of the rice, including plant height and root length, by approximately 14% and 40%, respectively. Compared to the control check group (CK), the adverse effects of salt stress on the growth status, leaf photosynthesis, and physiological/biochemical indices in the rice seedlings were alleviated in the GR24 treatment group. With increases in the GR24 concentration, the plant height and root length of the seedlings increased. The plant height in the groups treated with 1/2 Hoagland’s complete nutrient solution + 200 mM NaCl +1 μM GR24 (T4) and 1/2 Hoagland’s complete nutrient solution + 200 mM NaCl +5 μM GR24 (T5) were significantly different than the 1/2 Hoagland’s complete nutrient solution + 200 mM NaCl group (T1) (P < 0.05), and there were significant differences between the T5 and T1 groups in root length (P < 0.05).The chlorophyll content in the rice seedling leaves was significantly different between the T1 group and all other groups (P < 0.05). The net photosynthetic rate of the T1 group was not significantly different from the T2 group (P > 0.05). The transpiration rate, stomatal conductance, and intercellular CO_2_ concentrations showed the same trends as the net photosynthetic rate. The MAD, POD, and SOD activities were significantly increased by 68%, 60%, 14%, respectively, compared to the CK group (P < 0.01). When the GR24 concentration was 1 μM, the rice seedlings were resistant to the adverse effects of high salt stress. Therefore, the addition of proper concentrations of GR24 could improve the rice yield in saline-alkali land.

## Introduction

The world population maybe exceed eight billion by 2030 and global food production will become very important for the growing population^1^. However, various environmental factors and soil salinity have adversely affected plant growth and crop productivity, especially in arid and semi-arid regions. Statistical analyses have indicated that cultivated land with over 800 million hectares, which accounts for about 20% of the total cultivated land area on earth, has been adversely affected by salinity worldwide^2^. Salinity is one of the major stresses influencing the growth and yield of plants. To cope with salt stress, plants have evolved two main types of tolerance mechanisms based on either limiting the entry of salt by the roots or controlling its concentration and distribution^3^. Through complex physiological and biochemical reactions, salinity suppresses the growth and development of plants^4,5^. The research showed that reactive oxygen species may play an important role in salt stress. Plants tolerant to salt stress may evolve certain strategies, such as inducing lipid peroxidation; degrading nucleic acids and proteins; inactivating enzymes; and even causing necrocytosis in plants, to remove these reactive oxygen species, thus reducing their toxic effects^6^.

Many strategies have been used to improve the salt tolerance of crops. Beneficial microorganisms in soil can interact with the host plant to induce tolerance to abiotic stresses. Sadhana (2014) found that arbuscular mycorrhizal fungi could alleviate salt stress in host plants by enhancing the water absorption capacity, nutrient uptake, and accumulation of osmoregulators to increase the osmotic potential of the cells^7^. On the others, salt tolerance in crops through marker-assisted selection and genetic engineering. Genetic engineering has mainly focused on the effectors and regulatory genes involved in plant salt stress responses, especially on encoding enzymes, such as superoxide dismutase (SOD) and peroxidase (POD), antioxidant systems, and transcription factors^8^. Lin, *et al*. (2009) found that DWARF27, an iron-containing protein, was involved in the MAX/RMS/D pathway, in which D27 acts as a new member participating in the biosynthesis of strigolactones^9^. Moreover, it shows that phytohormones are involved in the physiological and biochemical reactions of abiotic stress, which play important roles in reducing damage caused by stressful environments^10,11^. Dar *et al*. (2017) reported that abscisic acid is a phytohormone that plays a pivotal role in the plant stress response to adverse environmental conditions by upregulating hormone-responsive transcription factors, kinases, and phosphatases and, in turn, enhancing plant adaptation to various abiotic stresses^12^. Li *et al*. (2018) reported that the plant hormone gibberellin could promote seed germination and also alleviate the inhibitory effects of salinity on seed germination. The study also reported that the action of gibberellin on rice seed germination in response to salinity inhibits seed germination by decreasing the bioactive gibberellin content. Furthermore, bioactive gibberellin deficiency inhibited seed germination by decreasing α-amylase activity via the downregulation of α-amylase gene expression^13^. Strigolactone, a carotenoid-derived terpene lactone, was initially isolated from a *Gossypium hirsutum* root culture solution by scientists in the 1960s^14^. In 2008, this substance was identified as a novel hormone that could remove and suppress the branch generation of higher plants^15^. Under limited nutrient conditions, strigolactones synthesized at the plant root promoted the growth of lateral roots and root hairs in an effort to increase the uptake of limited inorganic nutrients by the roots. Simultaneously, the stigolactones were transported to plant structures above the ground, which suppressed the generation of lateral buds or branches and reduced the requirements of the branches for inorganic nutrients^16,17^. However, its effects on the physiological and biochemical reactions of salt stress in rice are still unknown. Due to the low content of natural strigolactones in many plants, a series of strigolactone analogs, including GR5, GR7, and GR24, have been chemically synthesized, where GR24 had the highest activity^18^.

Generally, GR24 has been used as a positive control to study the biological activity of strigolactones, and it was found that strigolactones played specific roles in stress responses by regulating abiotic stresses. For example, exogenous GR24 played specific regulating roles in the drought resistance and salt tolerance of *Arabidopsis thaliana*^19,20^.

In this study, the effects of exogenous GR24 on photosynthesis, as well as its related physiological and biochemical reactions, in salt-stressed rice seedlings were studied. The relationship between exogenously added GR24 and the growth status of rice seedlings under salt stress and the related parameters of photosynthesis and enzymatic activity in rice seedling cells were investigated to confirm the optimal amount of GR24 for reducing damage to rice seedlings under salt stress and provide a theoretical foundation for the expansion of rice planting areas.

## Results and Discussion

Experiments with the control check (CK) and 1/2 Hoagland’s complete nutrient solution + 200 mM NaCl treatment (T1) groups showed that 200 mM NaCl significantly suppressed the growth of rice seedlings (P < 0.05), including the plant height, root length, stem leaf weight, and root weight. Alhasnawi *et al*. (2016) evaluated rice seedling tolerance under 200 mM salinity stress by studying the physiological and biochemical parameters of polysaccharides (β-glucan)^21^. Therefore, 200 mM NaCl was selected as the salt stress condition for this study. A preliminary experiment showed that 48 hours was the optimal time to study salt stress. The seedlings were treated with different concentrations of GR24 and the results are shown in Table 1. Salt stress significantly suppressed the growth of the aboveground and underground plant parts. However, synthetic strigolactone GR24 relieved the suppression of rice plant growth by salt stress. The 1/2 Hoagland’s complete nutrient solution + 200 mM NaCl + 1 μM GR24 (T4) and 1/2 Hoagland’s complete nutrient solution + 200 mM NaCl + 5 μM GR24 (T5) groups had significantly different plant heights compared to the T1 group (P < 0.05), but there were no significant differences between the 1/2 Hoagland’s complete nutrient solution + 200 mM NaCl + 0.1 μM GR24 (T2), the 1/2 Hoagland’s complete nutrient solution + 200 mM NaCl + 0.2 μM GR24 (T3), and the T1 groups. There were significant differences in root length between the T5 group and the T1 group (P < 0.05), but the T2, T3, and T4 groups were not significantly different than the T1 group. The biomass of the stem leaves in the T3, T4, and T5 groups was significantly different compared to the T1 group (P < 0.05), but there were no significant differences between the T2 and T1 groups. There were significant differences in root biomass between the T1 and all other groups (P < 0.05). When the GR24 concentration reached over 1 μM, the plant height was similar to that of the CK group, whereas the biomass was slightly higher than the CK group. However, the root length growth status in all groups was weaker than in the CK group. These results may be related to the fact that strigolactones are an ancient and major class of endogenous plant growth regulators, which can regulate the plant salt stress response by sensing and signaling. A previous study identified a strigolactone signaling pathway, including endocytosis of the PIN-FORMED1 auxin efflux carrier and transcript accumulation of the BRANCHED1 transcription factor^22^, and then found that strigolactone signaling acted through the degradation of D53 proteins for shoot branching in rice^23^. Therefore, with increasing GR24 concentrations, root biomass significantly increased, which may be related to the fact that strigolactone suppressed the growth of the aboveground plant parts and improved the root growth.

**Table 1:** Effects of GR24 on plant height, root length, and biomass of rice seedlings under salt stress.

Experimental No.	Plant height (cm)	Root length (cm)	Stem leaf wet weight (g plant^−1^)	Root wet weight (g plant^−1^)	Stem leaf dry weight (g plant^−1^)	Root dry weight (g plant^−1^)
CK	26.25 ± 4.90^ab^	13.63 ± 2.93^a^	1.77 ± 0.08^c^	0.54 ± 0.03 ^cd^	0.34 ± 0.01^e^	0.059 ± 0.002^d^
T1	22.58 ± 0.43^b^	7.80 ± 0.24^c^	1.65 ± 0.06^c^	0.36 ± 0.03^e^	0.40 ± 0.03^c^	0.062 ± 0.004^d^
T2	25.25 ± 1.32^ab^	8.38 ± 0.48^bc^	1.74 ± 0.07^c^	0.47 ± 0.02^d^	0.53 ± 0.02^b^	0.098 ± 0.003^c^
T3	26.16 ± 1.02^ab^	8.88 ± 0.63^bc^	1.92 ± 0.05^b^	0.58 ± 0.03^bc^	0.62 ± 0.03^a^	0.127 ± 0.002^a^
T4	28.43 ± 1.21^a^	9.03 ± 0.88^bc^	2.25 ± 0.05^a^	0.65 ± 0.04^b^	0.67 ± 0.03^a^	0.131 ± 0.003^a^
T5	27.65 ± 0.91^a^	10.63 ± 1.20^b^	2.19 ± 0.06^a^	0.86 ± 0.07^a^	0.64 ± 0.01^a^	0.114 ± 0.006^b^

Note: Different characters represent the achieved significance level between GR24 treatments (*P* < 0.05).

The strigolactone treatment group had increased plant height and root length compared to the high-salt stress treatment group. The increased root system of the rice seedlings in the treatment group was conducive to absorbing more nutrients and water. The increased plant height suppressed the growth of lateral buds and was conducive to full photosynthesis in the rice, which gave the rice a much stronger competitive advantage under adverse stress. Strigolactone may play important regulatory roles in the growth of aboveground branches and underground root systems in plants (Table 1).

**Figure 1:**
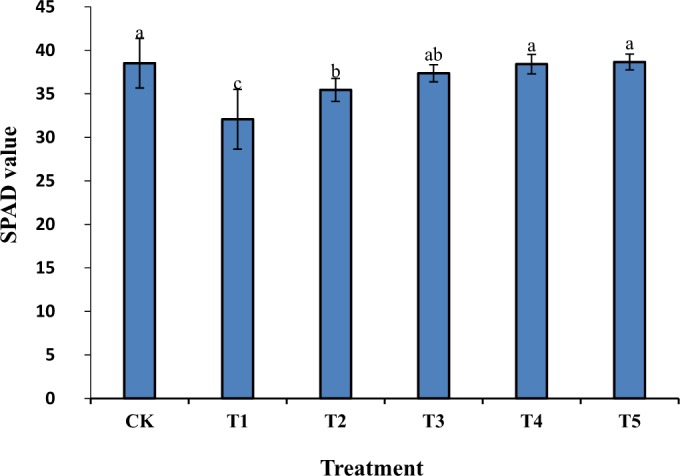
Effects of different concentrations of GR24 on chlorophyll content in rice seedling leaves under salt stress.

The results in Fig. 1 show that the chlorophyll content in the rice seedling leaves in the T1 group significantly was decreased compared to the CK group (P < 0.05), and the SPAD value decreased by 16%. However, there were significant differences between the T1 group and all other groups (T2, T3, T4, and T5) treated with different concentrations of GR24 (P < 0.05), in which the SPAD values increased by 11%, 16%, 20%, and 20%, respectively. When the GR24 concentration reached 0.2 uM, the effects of salt stress on the chlorophyll content in rice seedling leaves were counteracted. When the GR24 concentration was more than 1 μM, there were no significant changes in chlorophyll content. The atmospheric CO_2_ was decreased due to enhanced stomatal closing, and reductions in the consumption of NADPH through the Calvin cycle. When ferrodoxin is enhanced through photosynthetic electron transfer, it may be converted from PS-I to oxygen to form superoxide radicals through the Mehler Reaction, which induces chain reactions that generate more oxygen radicals^24^. This is reflected in the physiological parameters, such as the transpiration rate, stomatal conductance, and intercellular CO_2_ concentrations.

**Figure 2:**
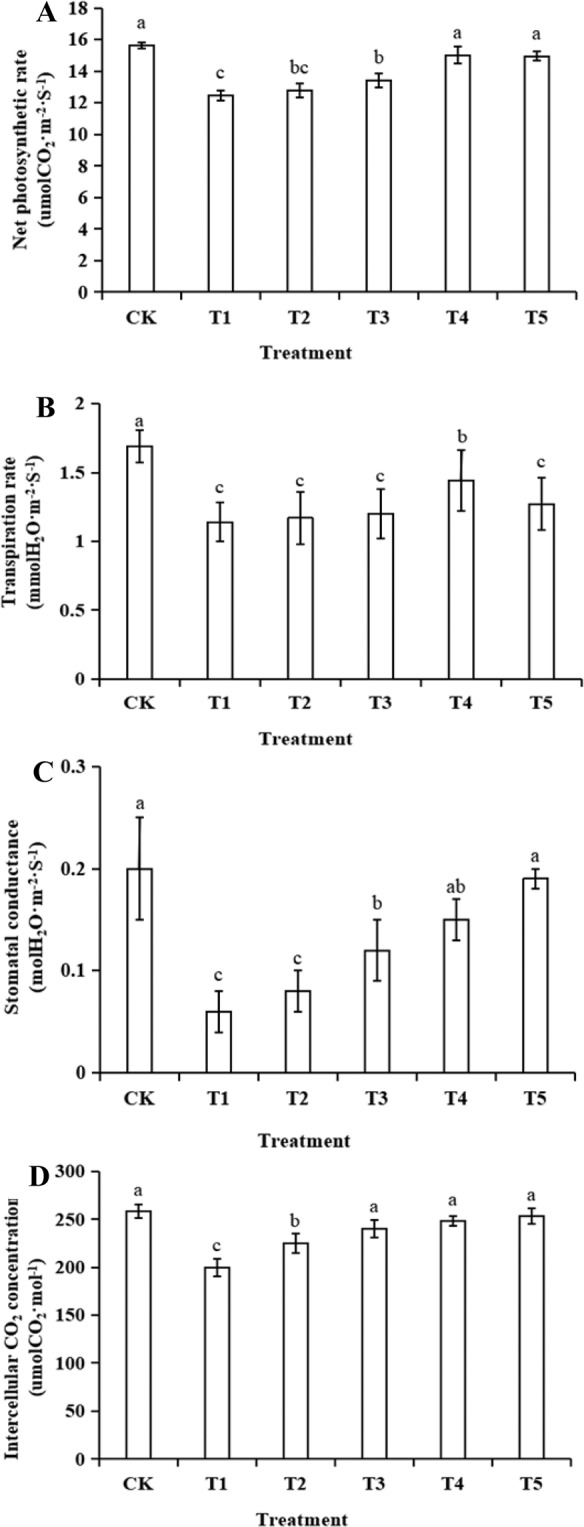
Effects of different concentrations of GR24 on photosynthetic parameters in rice seedling leaves under salt stress.

The results in Fig. 2 show that the net photosynthetic rate in the T1 group decreased by 20% compared to the CK group, and the differences were significant (P < 0.01). However, there were no significant differences between the T2 group and the T1 group, but there were significant differences between T2 and the other groups (P < 0.05). The transpiration rate, stomatal conductance, and intercellular CO_2_ concentrations showed the same trends as the net photosynthetic rate. The use of GR24 could increase the photosynthetic parameters of the leaves exposed to salt stress. Simultaneously, the parameter values were also related to GR24 concentrations. For example, the transpiration rate in the T1 group decreased by 32% compared to the CK group, the stomatal conductance decreased by 60%, and the intercellular CO_2_ concentration decreased by 22%. With an increase in GR24 concentration, the effects caused by salt stress recovered at 1 μM, and the increase in concentration had no significant effect on photosynthesis.

**Figure 3:**
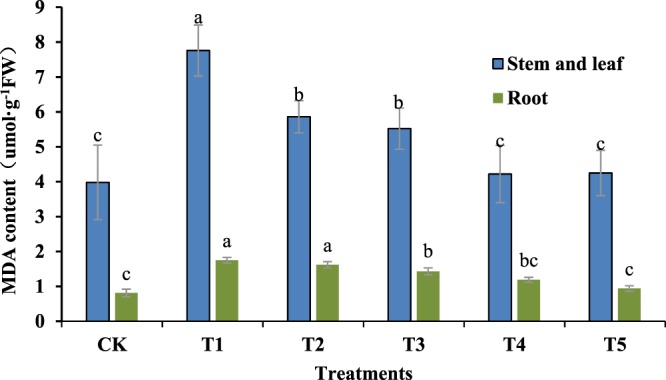
Effects of different concentrations of GR24 on MDA content in rice seedlings under salt stress.

**Figure 4:**
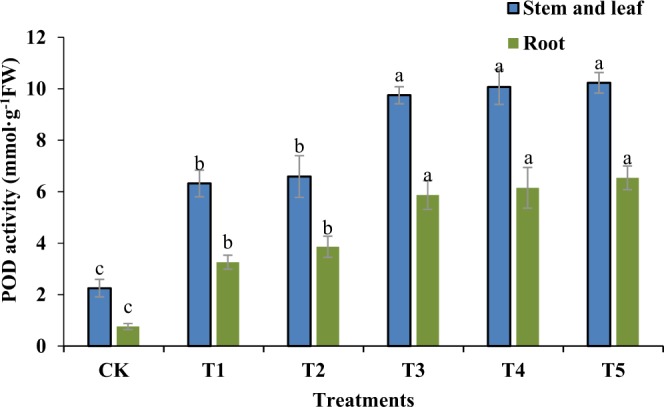
Effects of different concentrations of GR24 on POD activity in rice seedlings under salt stress.

Malondialdehyde is one of the main products of membrane lipid peroxidation. Its content is an important index that reflects the level of cell membrane lipid peroxidation. Figure 3 shows that salt stress significantly increased the malondialdehyde content in stem leaves and seedling roots. Compared to the CK group, the highest increase in the malondialdehyde content reached 68%, and the difference was significant (P < 0.01). This indicates that a salt concentration of 200 mM, produced much greater membrane lipid peroxidation in the Jinongda 667 seedlings. However, when GR24 was used to treat salt-stressed rice seedlings, the malondialdehyde content in the aboveground plant parts significantly decreased. GR24 had a stronger effect on reducing the malondialdehyde content. When the concentration was higher than 1uM, the effects diminished. For roots, the trend in variation was consistent with the aboveground plant parts. When the GR24 concentration reached 5 μM, the malondialdehyde content was lowest, and the content decreased to 46% in the salt-stress treated group, which had the same effects as 1 uM GR24 on the aboveground plant parts. The results in Fig. 4 show that with increasing concentrations of GR24, the POD enzymatic activity also increased in either the aboveground or underground plant parts. Compared to the CK group, all other groups showed significant differences (P < 0.01), and the enzymatic activity increased by over 60%. However, there were no significant differences between the T1 and T2 groups. Compared to the T1 group, the T3, T4, and T5 groups were significantly different (P < 0.05).

**Figure 5:**
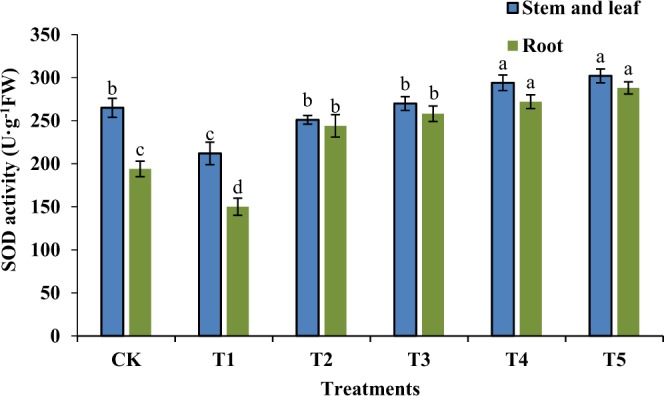
Effects of different concentrations of GR24 on SOD activity in rice seedlings under salt stress.

SOD is an important enzyme in systems that scavenge superoxide free radicals. When plants are stressed by their external environments, the enzymatic activity significantly decreases. As shown in Fig. 5, under salt stress, the SOD activity in the stem leaves and roots of the rice seedlings significantly decreased compared to the normal treatment group, and there were significant differences between the CK group and the T1 group (P < 0.01). GR24 treatment efficiently suppressed the decrease in SOD activity, whereas the SOD activities in the T2 and CK groups were the same. However, with increasing GR24 concentrations, the SOD activity was further enhanced. The highest increase was 14% compared to the normal treatment group. Compared to the T1 group, all differences were significant (P < 0.05). This indicated that GR24 could efficiently reduce the accumulation of *in vivo* superoxide free radicals resulting from salt stress, relieving the cellular damage caused by the peroxidation of reactive oxide species in order to maintain the normal growth of the rice seedlings. These results are consistent with a previous study by Jamil *et al*. (2014) that reported that plant-derived smoke solutions contained strigolactones precursors that significantly increased the germination percentage (23.3%) and seedling vigor of smoke-primed seeds compared to non-smoke-primed seeds at high salt concentrations^25^. Alhasnawi *et al*. (2016) also showed a significant effect on rice plants from MDA, SOD, and POD in various polysaccharides concentrations^21^. As a type of novel plant hormone, the effects of strigolactones on resisting biotic and abiotic stressors (including nutrient deficiency, drought stress, and salt stress) have become a research topic of interest^26,27^^.^ The research by Ha *et al*. ^19^ found that under drought and high-salt stresses, the addition of exogenous strigolactones significantly increased the survival proportion and germination rate of strigolactone-synthesis mutants (max3 and max4), as well as response mutants (max2), enhanced the drought tolerance of wild plants, and verified the positive regulating roles of strigolactones in stress responses in *Arabidopsis thaliana*, which were consistent with the results of this study. Other research has shown that that under drought and high-salt stresses, the arbuscular mycorrhiza symbionts of lettuce plants affected the level of strigolactones in the root systems^28^. They also showed that under high-salt stress, during GR24 processing, the photosynthetic parameters in rice seedlings all significantly increased^28^. Lv and Zhang *et al*.^29,30^ found that under stressors, strigolactones could induce and regulate stomatal closure, and also explained the corresponding molecular mechanism for regulating stomatal closure.

## Conclusion

Under salt stress conditions, the addition of GR24 resulted in greater rice seedling growth, POD and SOD activity, and intercellular CO_2_ concentrations compared to the control group. This work revealed that GR24 could relieve the damage of high-salt stress in rice seedlings. Strigolactones also played a role in the parameters related to photosynthesis, including the rice seedling net photosynthetic rate, transpiration rate, stomatal conductance, and intercellular CO_2_ concentration. The effects of strigolactone were correlated with GR24 concentrations. In the physiological and biochemical indices, the changes in rice seedling MDA, POD, and SOD were related to strigolactone concentrations. GR24 treatment significantly decreased the MDA content, increased the enzymatic activities of POD and SOD, and reduced the salt stress damage to rice seedlings. Considering both the economic and growth rate of rice seedlings, the optimal GR24 concentration range was determined to be from 0.1–1 uM. A GR24 concentration of 1 uM enhanced the photosynthetic performance of microalgae. These findings provided an efficient approach to improving the salt tolerance of rice seedlings by GR24. The addition of optimal concentrations of GR24 may relieve the damage of salt stress in rice seedlings, improve the adaptability of rice to high-salt environments, and guarantee a relatively stable yield of rice in saline-alkali lands.

## Materials and Methods

### Tested materials

The tested rice variety was Jinongda 667 (derived from Wu Zhihai Research Group, College of Agriculture, Jilin Agricultural University), which is japonica rice planted conventionally in the Jilin province of China. It was bred by the sexual hybridization of Jinongda 7 as the maternal strain and Tong 88-7 as the paternal strain, and the offspring were selected by tolerance to high salinity. (China Rice Data Center, No: 20190008). The synthetic strigolactone analog (GR24) was purchased from the Dutch company, Chiralix. The other reagents were all analytical reagents that were purchased from the Sinopharm Chemical Reagent Co., Ltd.

### Rice cultivation

Jinongda 667 was used for rice cultivation. Plump rice seeds with consistent size were selected and sterilized by soaking in 5% NaClO for 10 minutes, then repeatedly washed in sterile water 3–5 times until clean. The seeds were soaked in sterile water for 48 hours in a 28 ^o^C incubator, and the water was removed. The seeds were placed in sterile Petri dishes. The cultures were incubated in the dark in a 28 °C biochemical incubator, and pre-germination was conducted in distilled water for 24 hours. Then, all seeds with the same buds were sown in three plastic nursery pots (120 mm × 150 mm) and cultured with water in a greenhouse for one week. The rice seedlings with uniform growth were then transferred into 1/2 Hoagland nutrient solution and the culture was continued for about two weeks. To reduce the risk of seedling death, the rice seedlings were transplanted into homemade PVC pots containing 200 mM NaCl for 48 h. GR24 was dissolved in a small amount of absolute ethanol, and then 1/2 Hoagland nutrient solution was used to adjust the volume to the required concentration. After treatment with 200 mM NaCl, and different concentrations of GR24 were added for seven days. Three replicates were conducted for each treatment. The growth conditions of the rice seedlings were 28 °C for 14 h in the light and 20 °C for 10 h in the dark. The light intensity was between 260 and 350 μmol·m^−2^·s^−1^, and the relative humidity was between 60% and 70%. The rice seedlings were harvested after seven days of treatment, and the corresponding indices were analyzed. The experimental design is shown in Table 2.

**Table 2:** Treatments of experiment.

Experimental No.	Treatment Conditions
CK	1/2 Hoagland’s complete nutrient solution
T1	1/2 Hoagland’s complete nutrient solution + 200 mM NaCl
T2	1/2 Hoagland’s complete nutrient solution + 200 mM NaCl + 0.1 μM GR24
T3	1/2 Hoagland’s complete nutrient solution + 200 mM NaCl + 0.2 μM GR24
T4	1/2 Hoagland’s complete nutrient solution + 200 mM NaCl + 1 μM GR24
T5	1/2 Hoagland’s complete nutrient solution + 200 mM NaCl + 5 μM GR24

## Measuring methods

### Determination of plant height and root length

After the addition of GR24 to the culture for seven days, the plant height and root length of the rice plants were measured. A ruler was used to measure six random plants from each treatment group. The average plant height and root length were determined. The average value of three replicates of the plant height and root length measurements was calculated and used as the measured value.

### Determination of biomass

After seven days of GR24 treatment, the rice seedlings were harvested. Scissors were used to separate the aboveground part from the underground part of the rice plant, and deionized water was used to clean the rice leaves and roots carefully. Six random plants from each treatment group were taken for the measurement of the fresh weights. The average fresh weight was calculated, and the average value of three replicates was calculated and used as the measured value.

### Determination of chlorophyll content

A Minolta SPAD 502 Chlorophyll Meter (Japan) was used to measure the soil and plant analyzer development (SPAD) value of the fully expanded leaves on the top of the plant to determine the chlorophyll content^31^. This instrument determined the chlorophyll content in leaves rapidly, accurately, and non-destructively. Six leaves were analyzed each time and the measurements were repeated three times. The average value of the three replicates was calculated.

### Determination of photosynthetic parameters

The net photosynthetic rate, stomatal conductance, intercellular CO_2_ concentration, and transpiration rate were measured using an LI-6400 portable photosynthesis system (LICOR, USA). The measurements were made between 9:00 and 11:00 A.M. The CO_2_ concentration in the leaf chamber was 400 μmol·mol^−1^, the air velocity was 500 μmol·s^−1^, the light intensity was 1000 μmol·m^−2^·s^−1^, the leaf temperature was 30 ± 1 °C, and the relative air humidity was between 70% and 80%. The data were automatically collected every three minutes and repeated at least six times.

### Determination of physiological indices

After seven days of growth, the rice seedling leaves and root systems were harvested, quickly frozen by liquid nitrogen, and stored in at −80 °C for the determination of each physiological and biochemical parameter. All physiological index measurements were repeated three times. The MDA content was determined by the thiobarbituric acid method, the guaiacol method was used to determine the POD activity, and the SOD activity was measured by nitroblue tetrazolium (NBT) reduction^32^.

#### Determination of MDA

Fresh plant tissue (0.5 g) was homogenized and extracted in 10 ml of 0.25% TBA made in 10% trichloroacetic acid (TCA)^32^. The extract was heated at 95 °C for 15 min and then quickly cooled on ice. After centrifugation at 5,000 × g for 10 min, the absorbance of the supernatant was measured at 532 nm. A correction for non-specific turbidity was performed by subtracting the absorbance at 600 nm. The lipid peroxidation level was expressed as nanomoles per gram of fresh weight by using an extinction coefficient of 155 mM cm^−1^.

#### Determination of POD activity

The antioxidant activity was determined using the protocol with some modifications. The leaf samples were ground with a mortar and pestle under chilled conditions in homogenization buffer. The homogenate was centrifuged at 10,000 × g for 20 min at 4 °C, and the supernatants were used for the enzymatic assays. The guaiacol POD activity was measured with guaiacol as the substrate in a total volume of 3 ml. The reaction mixture consisted of 50 mM potassium phosphate buffer (pH 6.1), 1% guaiacol, 0.4% H_2_O_2_, and enzyme extract. The increase in the absorbance due to the oxidation of guaiacol was measured at 470 nm. One unit of POD activity was defined as the OD_470nm_ value reduced by 0.01 in one minute.

#### Determination of SOD activity

Superoxide dismutase activity was assayed using the photochemical NBT method. The samples (0.5 g) were homogenized in 5 ml of extraction buffer consisting of 50 mM phosphate (pH 7.8). The 3 ml assay mixture contained 50 mM phosphate buffer (pH 7.8), 26 mM methionine, 750 µM NBT, 1 µM EDTA, and 20 µM riboflavin. The photoreduction of NBT (formation of purple formazan) was measured at 560 nm and an inhibition curve was generated using different extract volumes. One unit of SOD activity was defined as the volume of extract causing 50% inhibition in the photoreduction of NBT. Determination of the physiological indices was performed in triplicate and the results were averaged.

### Statistical analyses

The experimental data are represented as the average value ± standard deviation. Statistical analyses were performed using SPSS software (SPSS 2013). Duncan’s multiple range tests were employed to assess the significant differences between the different initial GR24 concentrations under the same other cultivation conditions. The significance threshold was set at a probability level of p = 0.05.
